# Acute acalculous cholecystitis in children due to EBV and cytomegalovirus infection: a rare case report and review of the literature

**DOI:** 10.3389/fped.2026.1808120

**Published:** 2026-06-22

**Authors:** Wenling Ding, Renchang Liu

**Affiliations:** 1 Department of Neonatology, Weifang Hospital of Traditional Chinese Medicine, Weifang, Shandong, China; 2 Department of Gastroenterology, Weifang Hospital of Traditional Chinese Medicine, Weifang, Shandong, China

**Keywords:** acute acalculous cholecystitis, child, cytomegalovirus, Epstein–Barr virus, infectious mononucleosis

## Abstract

Epstein–Barr virus (EBV) is a B-cophilic double-stranded DNA virus with strong susceptibility throughout the world. Among young children under 6 years old, primary EBV infections are mostly recessive, In older children, EBV infection often involves multiple systems, presents as infectious mononucleosis (50%), chronic active EBV Infection, hemophagocytic syndrome, tumor-related diseases and autoimmune diseases, etc. Cytomegalovirus (CMV) is another common pathogen causing infectious mononuclear cytopathic syndrome, which is prone to missed diagnosis in clinical practice. Acute cholecystitis in children is rare in clinical practice. Diseases with related cross-clinical manifestations are prone to missed diagnosis and misdiagnosis. We report the case of a 7-year-old boy with AAC occurring during IM caused by EBV and CMV coinfection. He presented with fever, cervical lymphadenopathy, abdominal pain, abnormal liver function, and gallbladder wall thickening without gallstones. After the diagnosis was clarified, antibiotics were discontinued and the patient improved with conservative treatment alone. This case highlights that EBV/CMV-associated AAC should be considered in children with IM who develop right upper abdominal pain, in order to avoid unnecessary antibiotic exposure and surgical intervention.

## Introduction

Epstein–Barr virus (EBV) is a human herpesvirus that infects B lymphocytes and is usually transmitted through saliva (1, 2). Infectious mononucleosis (IM), most commonly caused by EBV and less frequently by cytomegalovirus (CMV), is characterized by fever, pharyngitis, lymphadenopathy, hepatosplenomegaly, and atypical lymphocytosis (3–5). Although IM is usually self-limiting, hepatobiliary involvement may occur. Acute cholecystitis is relatively uncommon in children, and acute acalculous cholecystitis (AAC) is particularly rare (6). In pediatric patients, AAC may present with nonspecific abdominal pain, fever, and abnormal liver tests, which can mimic bacterial infection or a surgical abdomen. Viral-associated AAC has been increasingly reported, especially in association with EBV, but EBV and CMV coinfection with AAC remains very rare. Here, we describe a child with EBV and CMV coinfection who developed AAC during the course of IM. This case illustrates the importance of recognizing viral AAC in order to guide appropriate conservative management.

## Case report

A 7-year-old boy presented to our pediatric emergency department with a 4-day history of fever and severe abdominal pain. On admission, his vital signs were stable (temperature 37.5 °C, heart rate 105/min, blood pressure 90/65 mmHg, respiratory rate 20/min, oxygen saturation 98%) although he appeared uncomfortable. Physical examination showed bilateral cervical lymphadenopathy and right inguinal lymph node enlargement, as well as mild swelling and tenderness of both eyelids and temporal regions. The pharyngeal mucosa was congested. Abdominal examination revealed muscle tension below the xiphoid process and in the right upper quadrant, with a positive Murphy's sign. Family history was unremarkable.

Laboratory findings are summarized in Table 1 and etiological tests in Table 2. The patient had leukocytosis with marked lymphocytosis and atypical lymphocytes, elevated transaminases, mild hyperbilirubinemia, and characteristic T-cell subset changes. Serological and molecular testing showed positive EBV VCA IgM, positive EBV DNA, and positive CMV IgM, supporting EBV and CMV coinfection.

**Table 1:** Laboratory findings on admission.

Examination Item	Test Result	Normal Reference Range
White Blood Cell	18.56 × 10^9/L	4.0∼12.0 × 10^9/L
Neutrophil Percentage	12.4%	50%∼70%
Red Blood Cell	4.42 × 10^12/L	4.0∼5.5 × 10^12/L
Hemoglobin	120 g/L	110∼150 g/L
Platelet	157 × 10^9/L	100∼300 × 10^9/L
Manual Classification: Lymphocyte	70%	20%∼40%
Manual Classification: Atypical Lymphocytes	30%	0%∼2%
ESR	3.63 mg/L	0∼20 mm/h
Alanine Aminotransferase	280.5 U/L	5∼40 U/L
Aspartate Aminotransferase	272.2 U/L	5∼40 U/L
Total Bilirubin	21.2 μmol/L	3.4∼17.1 μmol/L
Direct Bilirubin	10.2 μmol/L	0∼6.8 μmol/L
Serum Creatinine	44 μmol/L	27∼62 μmol/L
CK-MBCr	26.9 U/L	0∼25 U/L
Procalcitonin	0.545 ng/mL	0∼0.5 ng/mL
Immunoglobulin A/G/M	IgA: 3.72 g/L, IgG: 13.1 g/L, IgM: 1.25 g/L	IgA: 0.7∼3.8 g/L; IgG: 7∼16 g/L; IgM: 0.5∼2.1 g/L
D-dimer	0.89 mg/L	0∼0.5 mg/L
Lymphocyte Subpopulation: Absolute Lymphocyte Count	13,232/uL	1,500∼4,000/uL
Lymphocyte Subpopulation: Total T Lymphocyte Count	12,087/uL	800∼2,800/uL
Lymphocyte Subpopulation: Tc/Ts Cell Count	10,381/uL	400∼1,600/uL
Lymphocyte Subpopulation: CD3	91.46%	60%∼80%
Lymphocyte Subpopulation: CD3 + CD4+ (Th Cells)	7.49%	30%∼50%
Lymphocyte Subpopulation: CD3 + CD8+ (Tc/Ts Cells)	78.54%	20%∼40%
Lymphocyte Subpopulation: CD4/CD8 (Th/Ts)	0.10	1.2∼2.4

**Table 2:** Etiological investigations on admission.

Etiological examination	Test Result	Normal Reference Range
Mycoplasma Pneumoniae Antibody	Negative	Negative
Antistreptolysin O	6U/mL	0∼200 IU/mL
EBV VCA IgM	17.8 AU/mL	0∼10 AU/mL
EBV EA IgG	2.88 AU/mL	0∼10 AU/mL
EBV VCA IgG	8.37 AU/mL	0∼10 AU/mL
EBV NA IgG	0.01 AU/mL	0∼10 AU/mL
CMV IgM	25.824 AU/mL	0∼10 AU/mL (negative)
EB DNA	Positive (+)	Negative (-)
Quantitative Hepatitis B Five-Item: Anti-HBs	94.13 mIU/mL	≥10 mIU/mL (protective level)
Hepatitis A Antibody	Negative	Negative
Hepatitis C Antibody	Negative	Negative
Other Respiratory Pathogens	Negative	Negative
Blood culture	Negative	Negative

**Figure 1:**
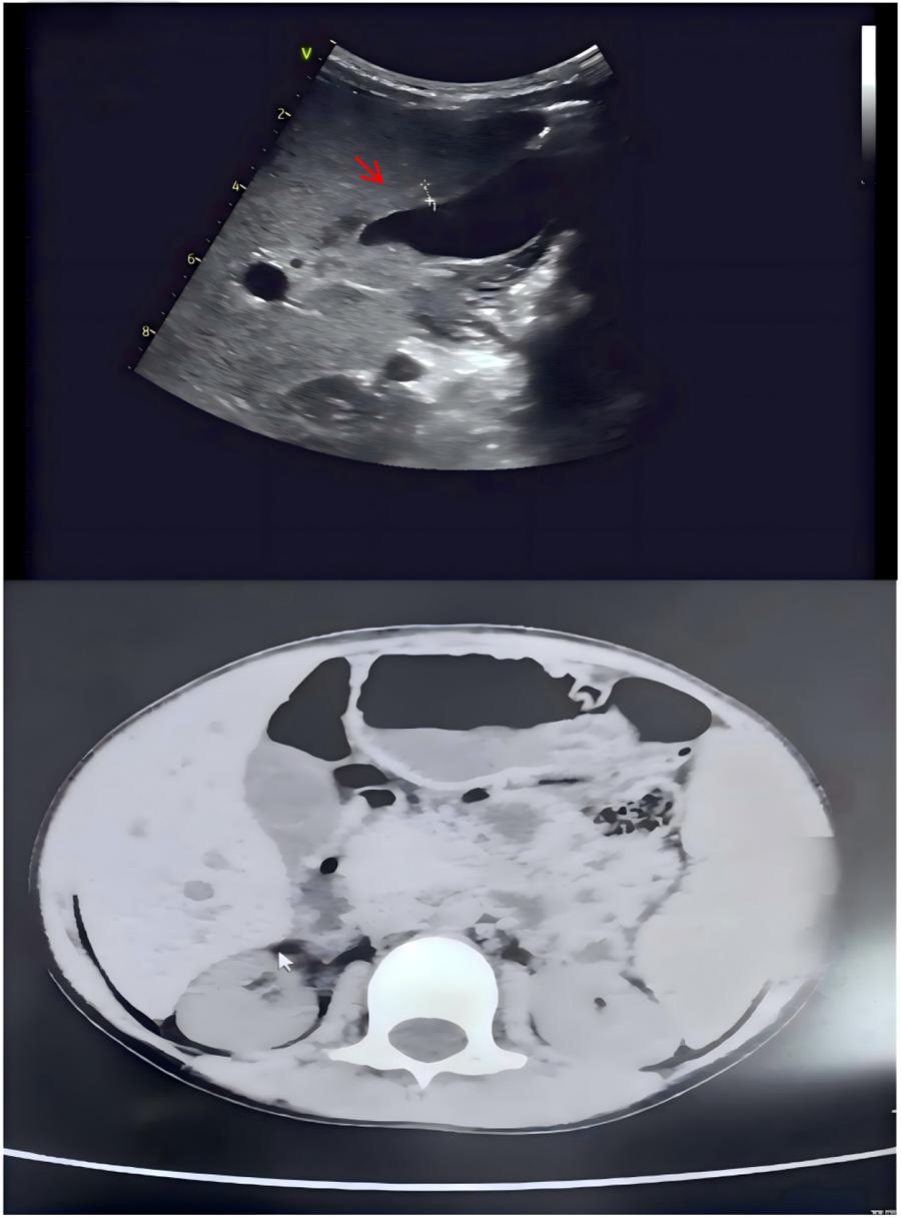
Abdominal ultrasound findings in the acute phase (May 28, 2025). Ultrasound showed marked gallbladder wall thickening with edematous/honeycomb-like change and no gallstones, consistent with acute acalculous cholecystitis. Splenomegaly was also present.

On May 28, 2025, abdominal ultrasound showed marked gallbladder wall thickening without gallstones, consistent with acute acalculous cholecystitis (AAC) (Figure 1). The imaging findings also suggested splenomegaly and abdominal lymphadenopathy, in keeping with systemic viral infection.

At the initial surgical assessment, acute cholecystitis was suspected, and broad-spectrum antibiotics were started while surgery was considered if the abdominal signs worsened. However, further evaluation suggested that the gallbladder involvement was secondary to infectious mononucleosis rather than primary bacterial disease. The diagnosis was based on the combination of fever, pharyngeal congestion, generalized lymphadenopathy, atypical lymphocytosis, elevated liver enzymes, positive EBV VCA IgM, detectable EBV DNA, positive CMV IgM, and ultrasound evidence of gallbladder wall thickening without gallstones. Taken together, these findings supported a diagnosis of infectious mononucleosis due to EBV and CMV coinfection complicated by acute acalculous cholecystitis. Antibiotics were therefore discontinued on the second hospital day.

The patient was managed conservatively with fasting, intravenous fluids, antispasmodic and analgesic treatment, liver-protective therapy, and antiviral treatment with acyclovir. His fever resolved and abdominal pain improved markedly by the third hospital day. Repeat ultrasound showed a reduction in gallbladder wall thickening, and Murphy's sign became negative. Oral feeding was gradually resumed. Continued clinical and radiological improvement supported the decision to avoid surgical intervention.

**Figure 2:**
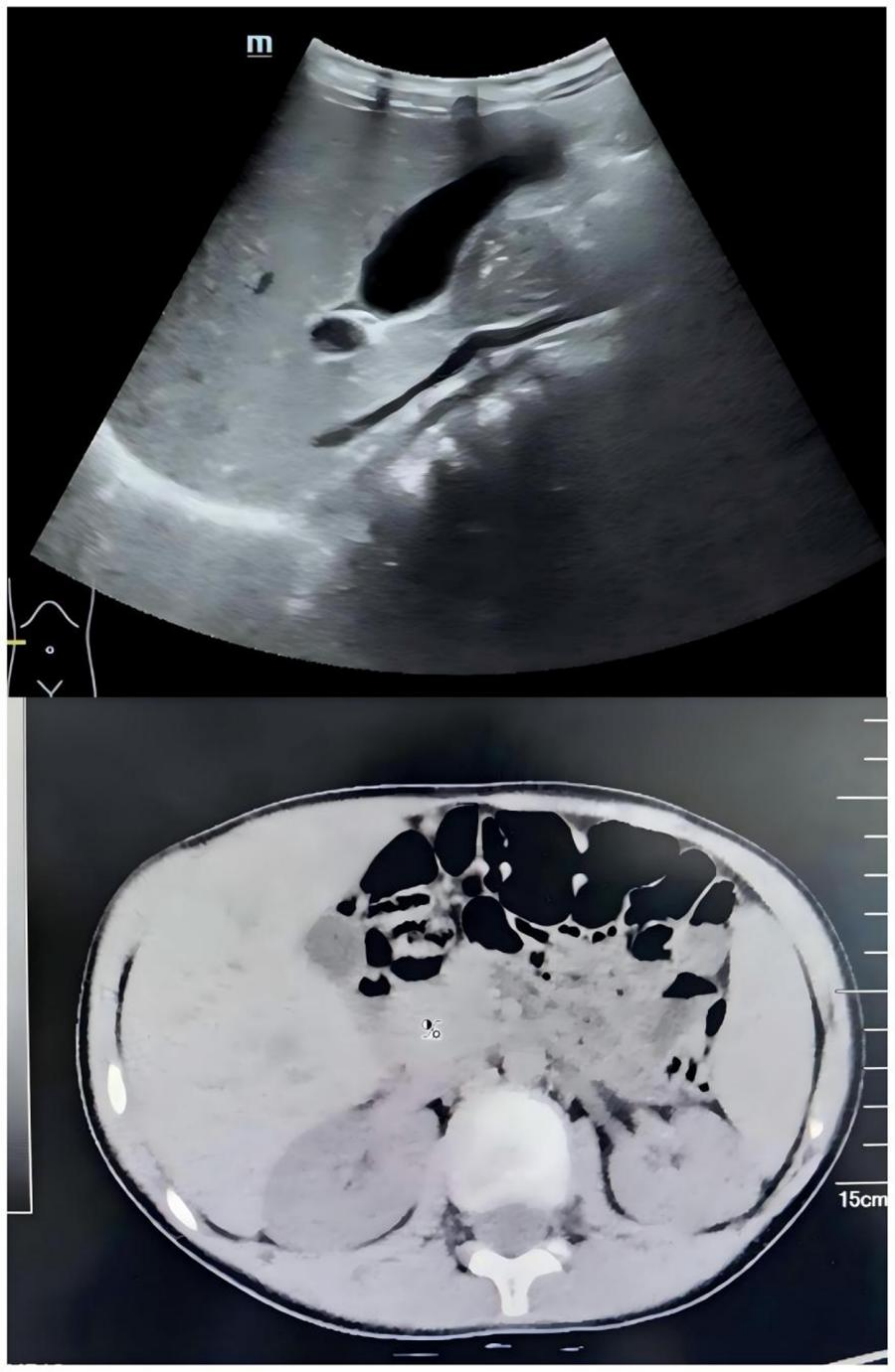
Follow-up abdominal ultrasound findings during recovery (June 6, 2025). Follow-up imaging demonstrated near-complete resolution of gallbladder wall thickening and improvement in splenomegaly, in parallel with clinical recovery.

After the acute abdominal symptoms had already improved, the child developed mild upper respiratory tract symptoms, including low-grade fever and rhinorrhea, during the later recovery phase. These symptoms appeared after the cholecystitis-related pain had substantially resolved and improved with symptomatic treatment alone. A subsequent ultrasound on June 6 demonstrated normalization of the gallbladder and partial regression of splenomegaly (Figure 2). The patient was advised to avoid strenuous activity and to return for follow-up.

## Discussion

AAC is an uncommon complication in children and may be difficult to diagnose because its presentation can overlap with both systemic viral illness and surgical abdominal disease (6). In previously reported pediatric cases associated with EBV infection, the most frequent findings include fever, abdominal pain, lymphadenopathy, hepatosplenomegaly, elevated liver enzymes, and gallbladder wall thickening without gallstones. Our patient presented with the same constellation of findings, including fever, right upper quadrant tenderness, cervical lymphadenopathy, splenomegaly, abnormal liver biochemistry, and ultrasonographic gallbladder wall thickening. This close clinicoradiological correlation with published cases strongly supports the diagnosis of viral AAC complicating infectious mononucleosis.

EBV is the leading cause of infectious mononucleosis, whereas CMV is another well-recognized cause of mononucleosis-like syndrome (3–5). Several studies have suggested that EBV/CMV coinfection may be associated with more prominent hepatic involvement and a more intense systemic inflammatory response than EBV infection alone (7–9). In our patient, the marked elevation of alanine aminotransferase and aspartate aminotransferase, mild hyperbilirubinemia, splenomegaly, and substantial expansion of CD8+ T lymphocytes were all in keeping with this pattern. The markedly reduced CD4/CD8 ratio was also consistent with the T-cell activation profile commonly reported in acute infectious mononucleosis and may have been accentuated by coinfection.

The diagnosis in this case was based on the integration of clinical, laboratory, microbiological, and imaging findings. The presence of fever, pharyngeal congestion, lymphadenopathy, atypical lymphocytosis, positive EBV VCA IgM, detectable EBV DNA, and positive CMV IgM supported the diagnosis of infectious mononucleosis due to EBV and CMV coinfection. At the same time, abdominal ultrasound demonstrated gallbladder wall thickening without stones, which is a typical feature of AAC. Importantly, serial imaging showed rapid improvement in parallel with clinical recovery, further supporting a transient inflammatory process related to the underlying viral infection rather than primary bacterial cholecystitis.

This diagnostic interpretation directly influenced management. At initial surgical review, broad-spectrum antibiotics and possible operative treatment were considered. However, once the overall picture was recognized as viral infectious mononucleosis complicated by AAC, antibiotics were discontinued and the patient was managed conservatively. He improved promptly with supportive care, and follow-up ultrasound confirmed resolution of gallbladder wall edema. This course is in agreement with previous reports showing that EBV-associated AAC in children is usually self-limited and can often be managed without surgery, provided that complications such as perforation, empyema, or persistent obstruction are excluded.

The exact pathogenesis of AAC in EBV- or CMV-associated infectious mononucleosis remains uncertain. Proposed mechanisms include direct viral involvement of the gallbladder epithelium, immune-mediated inflammation, cholestasis, and ischemic injury of the gallbladder wall (10–18). However, the possibility of a “bystander effect” should also be considered. In this context, the bystander effect refers to a situation in which EBV or CMV infection is present during the acute illness, but gallbladder inflammation is not caused by direct viral injury itself; instead, it may arise secondarily from systemic immune activation, transient bile stasis, dehydration, or nonspecific inflammatory responses associated with the underlying infection. Thus, detection of EBV- or CMV-related markers does not by itself prove that these viruses are the direct cause of AAC.

In our patient, several findings support a clinically meaningful association between EBV/CMV coinfection and AAC, including the typical features of infectious mononucleosis, the absence of gallstones, negative blood culture, compatible ultrasonographic findings, and rapid resolution of both symptoms and gallbladder wall thickening with conservative management alone. Nevertheless, because histopathological confirmation and direct virological examination of gallbladder tissue were not available, a bystander effect cannot be completely excluded. This distinction is important, because it emphasizes that viral-associated AAC should be interpreted cautiously as a clinicoradiological diagnosis rather than definitive proof of direct viral invasion.

From a practical clinical standpoint, however, the most important issue is early recognition of this entity. Whether gallbladder involvement reflects direct viral injury or an indirect bystander inflammatory process, awareness of this association may help clinicians avoid unnecessary antibiotic exposure, prolonged hospitalization, or unwarranted surgical intervention.

Another important point in this case is the temporal sequence of symptoms. The patient initially presented with fever and abdominal pain, which led to concern for primary cholecystitis. Mild upper respiratory tract symptoms developed later during convalescence, after the abdominal findings had already improved. Clarifying this sequence helps avoid confusion in interpreting the disease course and supports the view that the AAC was part of the initial EBV/CMV-associated illness rather than a secondary complication of a later respiratory infection.

This report has several limitations. As a single case, it cannot establish a definitive causal relationship between EBV/CMV coinfection and AAC. In particular, the possibility of a bystander effect cannot be excluded, meaning that the viral infection may have triggered a systemic inflammatory milieu in which gallbladder involvement developed indirectly rather than through direct viral invasion. In addition, histopathological confirmation of viral involvement in the gallbladder was not available. However, the combination of typical infectious mononucleosis features, compatible ultrasonographic findings, exclusion of other common infectious causes, and rapid resolution with conservative treatment makes the diagnosis of virus-associated AAC highly plausible.

In conclusion, AAC should be considered in children with infectious mononucleosis who develop right upper quadrant abdominal pain, abnormal liver function, and gallbladder wall thickening without stones. Recognition of this association may help clinicians make an appropriate diagnosis, avoid unnecessary antibiotics or surgery, and select timely conservative management.

## Data Availability

The raw data supporting the conclusions of this article will be made available by the authors, without undue reservation.
